# Network-Based Bioinformatics Highlights Broad Importance of Human Milk Hyaluronan

**DOI:** 10.3390/ijms252312679

**Published:** 2024-11-26

**Authors:** Kathryn Y. Burge, Hua Zhong, Adam P. Wilson, Hala Chaaban

**Affiliations:** Department of Pediatrics, University of Oklahoma Health Sciences Center, Oklahoma City, OK 73104, USA; kathryn-burge@ouhsc.edu (K.Y.B.); hua-zhong@ouhsc.edu (H.Z.); adam-wilson@ouhsc.edu (A.P.W.)

**Keywords:** necrotizing enterocolitis, preterm infant, glycosaminoglycan, network analysis, bioinformatics, hyaluronan, human milk, antioxidant

## Abstract

Human milk (HM) is rich in bioactive factors promoting postnatal small intestinal development and maturation of the microbiome. HM is also protective against necrotizing enterocolitis (NEC), a devastating inflammatory condition predominantly affecting preterm infants. The HM glycosaminoglycan, hyaluronan (HA), is present at high levels in colostrum and early milk. Our group has demonstrated that HA with a molecular weight of 35 kDa (HA35) promotes maturation of the murine neonatal intestine and protects against two distinct models of NEC. However, the molecular mechanisms underpinning HA35-induced changes in the developing ileum are unclear. CD-1 mouse pups were treated with HA35 or vehicle control daily, from P7 to P14, and we used network and functional analyses of bulk RNA-seq ileal transcriptomes to further characterize molecular mechanisms through which HA35 likely influences intestinal maturation. HA35-treated pups separated well by principal component analysis, and cell deconvolution revealed increases in stromal, Paneth, and mature enterocyte and progenitor cells in HA35-treated pups. Gene set enrichment and pathway analyses demonstrated upregulation in key processes related to antioxidant and growth pathways, such as nuclear factor erythroid 2-related factor-mediated oxidative stress response, hypoxia inducible factor-1 alpha, mechanistic target of rapamycin, and downregulation of apoptotic signaling. Collectively, pro-growth and differentiation signals induced by HA35 may present novel mechanisms by which this HM bioactive factor may protect against NEC.

## 1. Introduction

Nutrients within human milk (HM) spur growth and development during infancy. Bioactive components within HM, such as lactoferrin, immunoglobulin, and HM oligosaccharides [1], provide healthy building blocks for colonization and maintenance of the gut microbiome and maturation of intestinal immune cells. Infants on an exclusively HM diet are exposed to 50% fewer enteric infections compared with infants fed formula [2,3]. Importantly, protection offered by HM extends to necrotizing enterocolitis (NEC) [4,5,6,7,8,9], a life-threatening intestinal inflammatory disorder afflicting primarily premature infants. The etiology of NEC is complex and multifactorial, and risk factors beyond prematurity, including antibiotic use [10], dysbiosis [11], and formula feeding [12], contribute to increased incidence and severity of the disease. Studies have indicated preterm infants exposed to even a partial bovine-based formula diet are susceptible to increased NEC morbidity and mortality [13], while very low birthweight infants fed a diet of at least 50% HM within the 2 weeks following birth are six times less likely to develop NEC [14]. Much of the protection afforded by HM feeding has been attributed to bioactive factors, but how NEC risk is altered in association with isolated HM bioactive factors, rather than the composite, remains incompletely understood.

Unlike bovine-based infant formula [15], HM is rich in hyaluronan (HA), an ubiquitous and unique, non-sulfated glycosaminoglycan (GAG) with repeating linear residues of D-glucuronic acid and *N*-acetyl-D-glucosamine [16]. A primary component of the extracellular matrix (ECM), HA is produced endogenously by membrane-bound HA synthases (HASs) 1–3 [17]. The molecular weight, mode of administration, and local tissue physiology largely dictate the pro- or anti-inflammatory properties of HA [18,19]. In general, high molecular weight (HMW, >800 kDa) HA synthesized by HAS enhances tissue hydration, has antioxidant properties, and inhibits endothelial cell growth [20]. Oligo (<10 kDa), low MW (LMW, 10–300 kDa), and medium MW (MMW, 300–800 kDa) HA, produced either via enzymatic or oxidative degradation [20], induce tissue healing and repair through activation of the innate immune system, induce growth factor secretion, and stimulate angiogenesis [20,21,22,23,24,25,26,27,28].

Concentrations of HA peak in colostrum and early HM during the first weeks after birth, suggesting a critical role for exogenous HA during intestinal development postnatally [29]. As the proximal small intestine lacks the enzymes required to degrade HA [30], HM HA likely reaches the distal small intestine intact. In animal models of colitis, oral provision of HA, either purified from HM or produced biosynthetically at an average MW of 35 kDa (HA35), protects against bacterial-induced disease through increased antimicrobial β-defensin and tight junction protein expression within the epithelium [31,32,33,34]. Notably, our group has also demonstrated HA35 is protective against in vitro mechanical injury of intestinal epithelial monolayers [35], as well as in two distinct animal models of NEC [36,37].

Most recently, we demonstrated that HA35 promotes maturation of the murine neonatal small intestine, increasing villous length, crypt depth, and secretory cell abundance within the ileal epithelium [38]. Initial functional analyses of the ileal transcriptome indicated enrichment of pathways associated with growth, proliferation, and survival, including human epidermal growth factor receptor 2 (HER2), extracellular signal-regulated kinase/mitogen-activated protein kinase (ERK/MAPK), mechanistic target of rapamycin (mTOR), and phosphatidylinositol 3-kinase/protein kinase B (PI3K/Akt) signaling. Here, we aim to utilize additional functional and network analyses to further characterize the anti-inflammatory and pro-growth ileal transcriptome induced by HA35 supplementation during the neonatal period. As NEC remains a therapeutically intractable disease, our goal of identifying mechanisms mediating HA35’s protective and maturational effects on the ileum could provide novel therapeutic targets for the disease.

## 2. Results

### 2.1. Oral HA35 Induces a Unique Transcriptional Response Within the Developing Ileum

**Figure 1:**
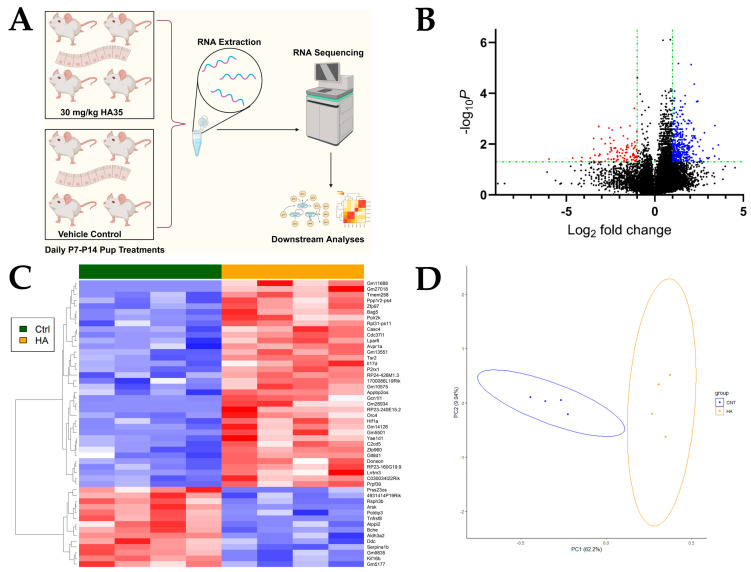
HA35 induces distinct transcriptional response during murine development. (**A**) Experimental design (*n* = 4 pups/group); (**B**) Volcano plot of DEGs *p*-value < 0.05; |log_2_FoldChange| > 1; upregulated with HA35 treatment, blue; downregulated with HA35 treatment, red; (**C**) Heatmap of top 50 DEGs; (**D**) PCA plot of DEGs. Abbreviations: P: postnatal day; RNA: ribonucleic acid; Ctrl: control; HA35: hyaluronic acid 35 kDa; PCA: principal component analysis; DEGs: differentially expressed genes.

Mouse pups were treated daily with 30 mg/kg body weight HA35 or vehicle control from postnatal day (P)7 to P14 (Figure 1A), a developmental timepoint corresponding with human intestinal development at 22–23 weeks’ gestation and a period of heightened susceptibility to NEC [39]. Bulk RNA-sequencing was performed on the distal ileum of pups at P14. From a total of 43,548 genes and using a *p*-value < 0.05 and |log_2_FoldChange| > 1, treatment with HA35 resulted in significant alterations in gene expression, with 249 differentially expressed genes (DEGs; 203 upregulated, 46 downregulated; Figure 1B). Dimensionality reduction in DEGs, the top 50 of which are illustrated in Figure 1C, from HA35-treated and control mice using principal component analysis (PCA) indicates pups cluster well by treatment (PC1, 62.2%; PC2, 9.04%; combined 71.24% variation explained; Figure 1D).

### 2.2. HA35 Induces Trend Toward Differentiation of Stromal, Mature Enterocytes, and Paneth Cells

**Figure 2:**
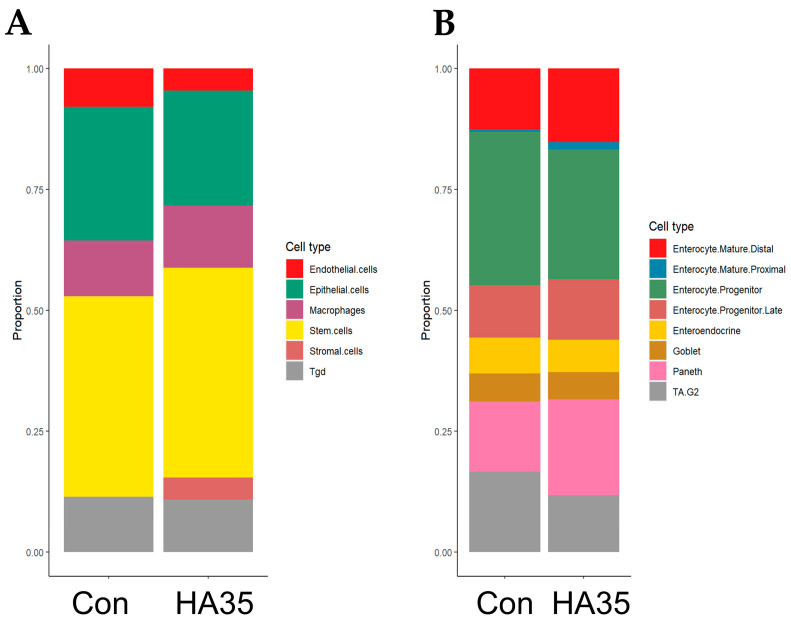
Oral HA35 may expand differentiated and stromal cell types. Mixed cell deconvolution using the Mouse Cell Atlas for (**A**) general intestinal and (**B**) intestinal epithelial cell types indicates a potential increase in stromal, Paneth, and mature enterocytes and progenitors, with diminished cycling TA, endothelial, and epithelial cells. Abbreviations: Con: Control; HA35: hyaluronic acid 35 kDa; Tgd: gamma delta T cells; TA.G2: transit-amplifying. Growth 2.

To evaluate the potential of HA35 to induce cell-type abundance changes in the distal ileum of developing pups, cell counts for general small intestine (Figure 2A; Figure S1) and intestinal epithelial-specific cell types (Figure 2B; Figure S2) were estimated using cellular deconvolution and gene expression profiles derived from the Mouse Cell Atlas scRNA-seq reference dataset [40]. Treatment with HA35 trended toward an enrichment in stromal cells, while endothelial and epithelial cell populations were expected to diminish in the developing ileum. In addition, Paneth, late enterocyte progenitor, mature proximal enterocyte, and mature distal enterocyte cell types within the intestinal epithelium showed indications of enrichment, potentially drawing from the reduced pool of cycling transit-amplifying progenitors (TA.G2). Altogether, HA35 may bolster differentiated secretory and absorptive cell types, as well as those within the mesenchymal compartment of the developing intestine.

### 2.3. Antioxidant and Growth Signaling Characterize Functional Intestinal Response to HA35

**Figure 3:**
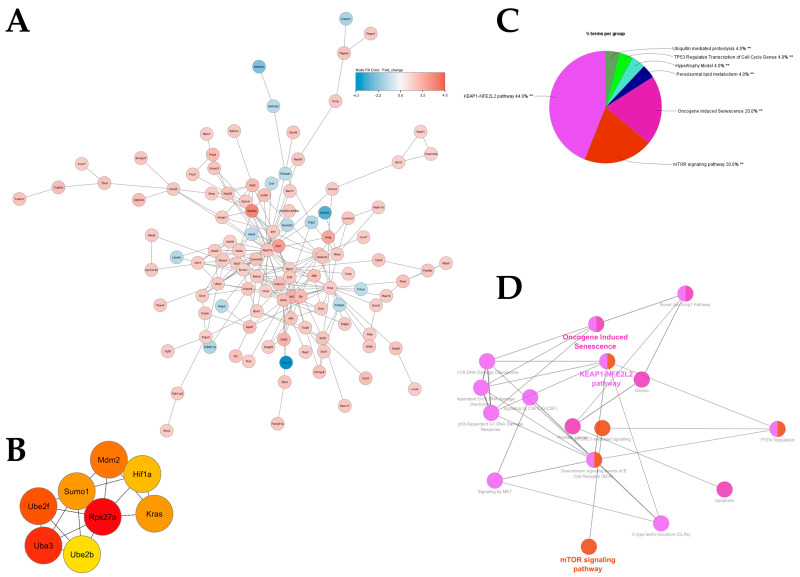
HA35 enriches signaling pathways involved in growth and antioxidant response in the developing murine intestine. (**A**) Largest PPI subnetwork demonstrating upregulated (red) and downregulated (blue) genes in HA35-treated pups compared with controls; (**B**) Top eight hub genes identified through CytoHubba (red = strongest associations); (**C**) ClueGO pie chart of GO term functional enrichment (% terms/group); ** FDR-adjusted *p* < 0.01; (**D**) ClueGO functional network (light pink: KEAP1-NFE2L2 pathway [44%]; red: mTOR signaling pathway [20%]; magenta: oncogene-induced senescence [20%]). Abbreviations: HA35: hyaluronic acid 35 kDa; PPI: protein–protein interaction; GO: gene ontology; FDR: false discovery rate; KEAP1-NFE2L2: kelch-like ECH-associated protein 1-nuclear factor erythroid 2-like 2; mTOR: mechanistic target of rapamycin; Ube2f: ubiquitin conjugating enzyme E2 F; Sumo1: small ubiquitin like modifier 1; Mdm2: mouse double minute 2; Hif1α: hypoxia inducible factor alpha; Rps27a: ribosomal protein S27a; Uba3: ubiquitin like modifier activating enzyme 3.

When comparing gene expression altered by HA35 treatment, we utilized DEGs to construct a protein–protein interaction (PPI) network, where nodes indicate discrete genes and edges represent connections between and among genes. The largest subnetwork (Figure 3A) was utilized for functional hub gene analysis, identifying *Rps27a* (ribosomal protein S27a), *Uba3* (ubiquitin like modifier activating enzyme 3), *Ube2f* (ubiquitin conjugating enzyme E2 F), *Mdm2* (mouse double minute 2), *Sumo1* (small ubiquitin like modifier 1), *Kras*, *Hif1α* (hypoxia inducible factor alpha), and *Ube2b* as genes driving connections within the PPI network (Figure 3B). The largest subnetwork was subjected to functional annotation using ClueGO, indicating enrichment of antioxidant (kelch-like ECH-associated protein 1-nuclear factor erythroid 2-like 2 [KEAP1-NFE2L2]) and growth (mechanistic target of rapamycin [mTOR]) pathways (Figure 3C) with HA35 treatment. The largest functional network based upon ClueGO analysis is depicted in Figure 3D.

**Figure 4:**
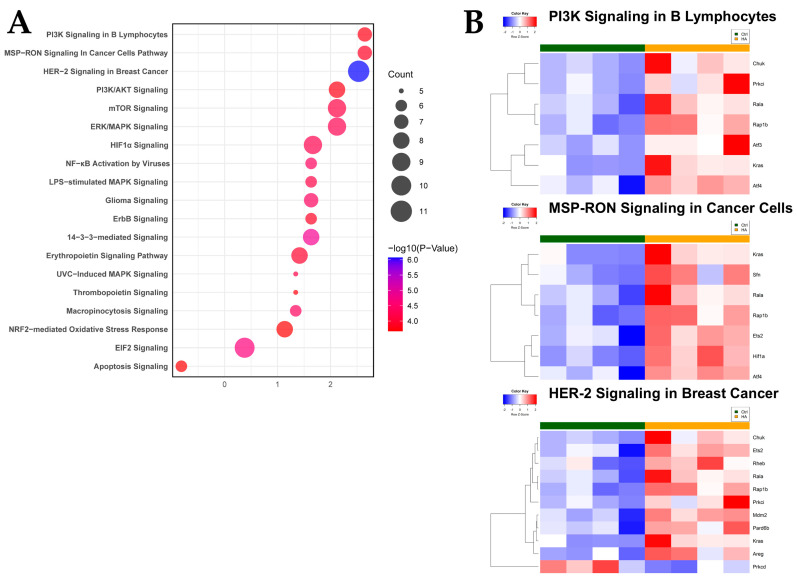
Enrichment analysis predicts upregulation of growth and downregulation of apoptosis signaling pathways following HA35 treatment of the developing intestine. (**A**) Bubble plot for IPA pathways; (**B**) Heatmaps for genes in PI3K, MSP-RON, and HER-2 signaling pathways. Abbreviations: HA35: hyaluronic acid 35 kDa; IPA: Ingenuity Pathway Analysis; PI3K/At: phosphatidylinositol-3-kinase/protein kinase B; MSP-RON: macrophage stimulating factor-RON; HER-2: human epidermal growth factor receptor 2; mTOR: mechanistic target of rapamycin; ERK/MAPK: extracellular signal-regulated kinase/mitogen-activated protein kinases; HIF-1α: hypoxia inducible factor-1 alpha; NF-κβ: nuclear factor kappa beta; LPS: lipopolysaccharide; ErbB: erythroblastic leukemia viral oncogene; UVC: ultraviolet light C; NRF2: nuclear factor erythroid 2-related factor 2; EIF2: eukaryotic initiation factor 2.

To further evaluate the functional canonical signaling pathways involved in the intestinal response to HA35, DEGs were uploaded to Ingenuity Pathway Analysis (IPA) for enrichment analysis. Interestingly, pathways associated with growth and antioxidant response were enriched in HA35-treated pups, including PI3K/Akt, MSP-RON (macrophage stimulating protein-RON), HER-2, mTOR, ERK/MAPK, 14-3-3-mediated, HIF-1α, EIF2 (eukaryotic initiation factor 2), ErbB (erythroblastic leukemia viral oncogene homologue) signaling, and NRF2-mediated oxidative stress response (Figure 4A; Figure S3), while apoptosis signaling was predicted to be downregulated. Heatmaps for expression of genes driving the three most highly enriched pathways associated with HA35 treatment, PI3K, MSP-RON, and HER-2 signaling, are provided in Figure 4B.

**Figure 5:**
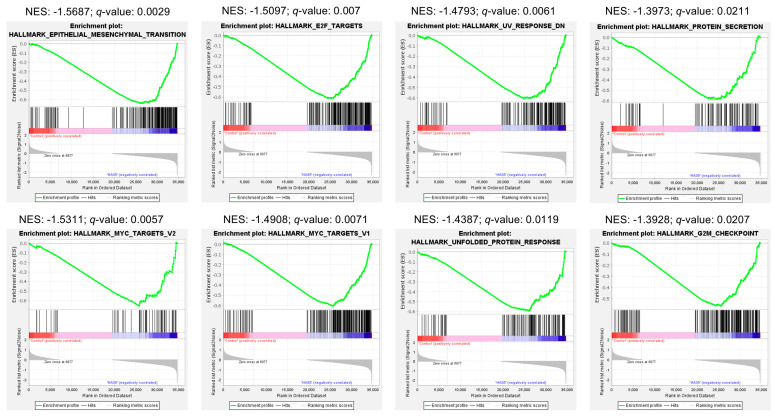
GSEA enrichment plots for top gene sets enriched (*q*-value < 0.05) in the distal ileum of HA35-treated pups versus controls. From top-left, left-to-right (gene overlap): Epithelial-mesenchymal transition (193); E2F targets (199); UV response down (144); Protein secretion (94); MYC targets V2 (58); MYC Targets V1 (197); Unfolded protein response (111); G2M checkpoint (195). Abbreviations: GSEA: gene set enrichment analysis; HA35: hyaluronic acid 35 kDa; NES: normalized enrichment score; UV: ultraviolet.

Finally, we performed gene set enrichment analysis (GSEA) to further explore functional effects of HA35 treatment on the developing murine intestine. GSEA revealed significant associations of epithelial mesenchymal transition, MYC targets, E2F targets, genes downregulated in response to UV radiation, unfolded protein response, protein secretion, and G2M checkpoint gene sets (Figure 5) with HA35 treatment. Core enrichment genes overlapping in multiple gene sets include genes associated with stromal cell and ECM expansion (e.g., *Ccn2* [cellular communication network factor 2], *Has2* [hyaluronic acid synthase 2], *Col1a1* [collagen type I alpha 1 chain], etc.), cell proliferation (e.g., *Pcna* [proliferating cell nuclear antigen], *Mki67* [marker of proliferation Ki-67], *Met*, *Kit*, *Myc*, *Egfr* [epidermal growth factor receptor], etc.), cell growth and survival (e.g., *Hif1a*, *Igfr2* [insulin-like growth factor 2 receptor], *Igfbp2/3* [insulin-like growth factor binding protein 2 and 3], *Vegfa* [vascular endothelial growth factor alpha], *Mapk14*, etc.), ubiquitination (e.g., *Ube2e1*, *Usp1* [ubiquitin specific peptidase 1], etc.), differentiation (e.g., *Wnt5a* [Wnt family member 5A], *Notch2* [notch receptor 2], etc.), and eukaryotic translational initiation factor genes (e.g., *Eif4e*, etc.). Overall, our data suggest HA35 promotes intestinal development during early neonatal life through a variety of pro-growth signaling pathways. In addition, the unexpected enrichment of pathways associated with antioxidant response and predicted reduction in apoptosis signaling may provide physiological protection against events predisposing preterm infants to NEC.

## 3. Discussion

NEC, a leading cause of morbidity and mortality in the neonatal period, is characterized by severe intestinal inflammation, resulting in abdominal distension, necrosis, and in severe cases, intestinal perforation, multi-organ failure, and death [41,42]. While the precise etiology of the disease remains unclear, NEC pathogenesis is multifactorial and incorporates intestinal immaturity [43], a dysregulated immune system [44], and an altered gut microbiome [45]. NEC onset typically occurs 2–3 weeks following birth [46], providing a crucial window of opportunity for clinical prevention. HM, particularly mother’s own milk (MOM), is thought to play a key role in protecting preterm infants from NEC through promoting maturation of the intestinal epithelial barrier, reducing intestinal permeability, and fostering development of a favorable microbiome [6,47,48,49,50,51].

Our group has published extensively on the protective effects of the HM HA mimic [32], HA35, both in vitro and in vivo. In the former [36], HA35 accelerates restitution and healing from a mechanical wound via cell proliferation at the wound edge and migration to the site of injury, while in the latter, HA35 reduces mortality, severity of intestinal injury, and systemic inflammation, in part through preservation of intestinal barrier function [36,37]. Importantly, in the unchallenged gut of postnatal mice, HA35 promotes intestinal development and maturation, increasing goblet and Paneth cell numbers, and deepening and lengthening crypts and villi, respectively [38]. Here, we sought to elucidate potential mechanisms by which HA35 induces maturational changes within the developing postnatal intestine.

### 3.1. Role of HA35 in Differentiation of the Intestinal Epithelium

The intestinal epithelium is spatially organized into villi for maximal nutritional absorption. Proliferative intestinal stem cells (ISCs) migrate from the crypt base toward the villi tips [52]. Fully differentiated enterocytes at villi tips undergo apoptosis and are shed into the lumen [53], generating a cycle of epithelial regeneration that renews the entire villus population within a matter of days. The critical barrier function of the intestine is maintained by expression of tight junctions, regulating the passage of luminal contents into the mucosa, as well as multiple layers of mechanical and chemical defense. Goblet cells secrete mucus, preventing direct microbial adherence to the epithelium [54]. Paneth cells chemically deter microbes via the release of antimicrobial peptides (AMPs) [55], and embedded immune cells within the submucosa functionally mature following stimulation by bacteria [56].

The intestinal epithelium of preterm infants is developmentally immature. Compared with those of infants born at term, goblet cells of preterm infants have a reduced capacity to generate mucins, resulting in a thinner mucus lining and increased susceptibility to bacterial translocation of the epithelium [43]. Preterm enterocytes secrete less detoxifying alkaline phosphatase, and Paneth cells produce less lysozyme and defensins, altogether allowing potential pathogens direct access to the single-cell barrier, with limited ability to combat bacterial toxins or prevent mucosal invasion.

Endogenous HA receptor binding during postnatal development induces Lgr5^+^ ISC proliferation, expansion of Paneth cell numbers, and enhanced crypt fission, with pericryptal macrophages implicated as the potential source of this signaling [57]. However, the role of HA35 in postnatal small intestinal development has not been as well-studied. Cell deconvolution of our bulk sequencing data predicts an increase in stromal cells and decreases in endothelial and epithelial cells. HA35-mediated expansion of the stromal cell lineage likely contributes to epithelial differentiation and maturation [58], enabling critical defense of the postnatal intestinal barrier. Within the epithelium, deconvolution predicted an increase in mature enterocytes, late enterocyte progenitors, and Paneth cells, and a reduction in enterocyte progenitors and cycling TA cells. Overall, the shift in cellular composition following HA35 treatment appears to align with increased maturation and enterocyte differentiation, and largely substantiates our prior in vivo immunohistochemical (IHC) studies indicating increased goblet and Paneth cells in these same animals [38]. Further, the increase in differentiated cell types likely draws from the reduced pool of cycling TA cells, the latter of which is associated with an increase in the ratio of secretory to absorptive enterocytes within the epithelium [59]. Interestingly, TA cell abundance represents a balance in capacity for cell differentiation or regeneration, and treatment with HA35 appears to shift the balance toward differentiation. Altogether, these observations indicate HA35 likely accelerates epithelial maturation and expansion of the stromal cell compartment without substantially altering immune cell abundances.

### 3.2. HA35 as an Inducer of the PI3K/Akt/mTOR Signaling Cascade

Signal transduction pathways associated with growth and epithelial proliferation are likely to influence NEC pathogenesis [60,61,62,63,64]. PI3K/Akt/mTOR signaling, fundamental to key cellular processes including protein synthesis and secretion, nutrient uptake, and glycolytic metabolism [65], is essential to meet the needs of a rapidly growing and differentiating intestine during the neonatal period [66]. Growth factors, cytokines, and toll-like receptor (TLR) ligands initiate mTOR activation, primarily through PI3K/Akt or ERK phosphorylation and inactivation of the repressor, tuberous sclerosis complex 1/2 (TSC1/2) [67]. Through integration of extracellular and intracellular signals, mTOR plays essential roles in cell growth, proliferation, differentiation, migration, and survival [68]. Importantly, the PI3K/Akt/mTOR signaling axis, when significantly disrupted, is often embryonically lethal. Global or conditional knockout of mTORC1 (mTOR complex 1), a key component of the mTOR pathway, results in intestinal epithelial defects, including ileal villus blunting and reduced numbers of Paneth and goblet cells [69], underscoring the importance of the pathway to intestinal development. Additionally, mTOR activation is vital for functional intestinal epithelial repair, demonstrated in models of ischemia-reperfusion injury, radiation-induced damage, and colitis [70].

The ability of HA to activate PI3K/Akt/mTOR signaling has been reported in monocytes, chondrocytes, and rod photoreceptors of the eye [71,72,73], providing plausibility for relevance of this pathway within the neonatal intestine. GSEA, IPA, and network analyses (Figure 3, Figure 4 and Figure 5) indicated treatment with HA35 activated PI3K/Akt/mTOR signaling within the developing murine ileum, supporting our prior IHC, quantitative polymerase chain reaction (qPCR), and Western blot indications of mTORC1 activation in these pups [38]. Enrichment of the PI3K/Akt/mTOR signaling axis suggests a mechanism by which HA35 supports robust intestinal development, potentially reducing susceptibility to complications like NEC in preterm infants.

### 3.3. Activation of HIF-1α and VEGF Signaling Through HA35

In the gut, HIF-1α has dual roles in intestinal development and prevention of hypoxic injury, such as that observed during NEC pathogenesis [74]. During gut development, activation of HIF-1α upregulates VEGF, promoting creation of new vasculature to ensure adequate oxygen delivery to rapidly expanding intestinal tissues [75]. Developmental angiogenesis is crucial in supporting epithelial cell growth, differentiation, and the formation of a functional intestinal barrier.

In the context of intestinal injury, HIF-1α activation is critical for initiating repair processes [76]. Upregulation of VEGF leads to enhanced angiogenesis, increasing the flow of blood to damaged tissues. Delivery of fresh nutrients and oxygen by new vasculature spurs regenerative processes, reduces further ischemic damage, and supports integrity of the intestinal barrier by inducing epithelial expression of tight junction proteins and antimicrobial peptides. HIF-1α also plays a protective role by preventing excessive mucosal apoptosis, further facilitating recovery of the intestinal epithelium.

Activation of ileal HIF-1α and VEGF signaling by HA35 correlates with similar processes in cancer biology, where overproduction of HA generates a positive feedback loop of increased metabolic demands, a shift toward glycolysis and PI3K/Akt-mediated glucose uptake, activation of HIF-1α, and a boost in endogenous HA synthesis [57,77,78]. This metabolic reprogramming also fuels upregulation of VEGF, promoting angiogenesis to enhance nutrient and oxygen delivery, facilitating both cancer progression and maintenance of cancer stem cell properties. The sum of these processes results in rapid cellular proliferation in increasingly hypoxic conditions. However, within the developing ileum, activation of HIF-1α and VEGF likely induces endothelial development, potentially protecting against mucosal injuries involving splanchnic hypoperfusion [79].

### 3.4. HA35 and the NRF2-Mediated Response to Oxidative Stress

The KEAP1-NRF2 pathway plays a crucial role in regulating response to oxidative stress through activation of a molecular system tuned to perturbations in cellular redox balance [80], orchestrating a network of inducible proteins in defense of homeostasis and preservation of gut integrity [81,82]. Under oxidative conditions, NRF2 dissociates from KEAP1 and translocates to the nucleus, where the protein binds antioxidant response elements (AREs), activating enzymes such as glutathione peroxidase and superoxide dismutase [83]. These antioxidant enzymes neutralize reactive oxygen species (ROS), minimizing oxidative damage within intestinal tissues, a process inherent to NEC pathogenesis [84]. Additionally, NRF2 activation reduces inflammation through inhibition of NF-κB, induction of epithelial tight junction protein (e.g., zonula occludens 1 [ZO-1], occludin) expression, and modulation of T-cell function [82,85,86]. Fragmentation of HMW HA by ROS is reportedly anti-inflammatory [22], and HA35 inhibits ROS production by neutrophils [87]. Activation of the anti-inflammatory KEAP1-NRF2 pathway by HA has been broadly reported in a number of tissues (e.g., [88]), and our functional enrichment analyses support extrapolation of these effects to HA35 within the developing neonatal ileum, implicating a role for HM HA35 in enhancing intestinal resilience, preventing oxidative damage, and mitigating risk of NEC.

Intriguingly, our network analyses revealed strong connections among the KEAP1-NFE2L2 pathway, oncogene-induced senescence, and mTOR signaling. Activating transcription factor 4 (*Atf4*) is strongly upregulated within the ileum of HA35-treated pups (Tables S1 and S2). A stress-induced transcription factor [89], ATF4 mediates crosstalk between the KEAP1-NRF2 and mTOR pathways, highlighting a potentially broader role for HA35 in regulating cellular adaptation to stress, promoting repair and survival, and hastening intestinal development, potentially contributing to prevention of NEC.

### 3.5. MSP-RON and Epithelial-Mesenchymal Transition (EMT) Signaling

Mucosal tissue damage is the primary injury incurred during NEC. Epithelial repair requires cell migration through EMT [90]. However, enterocytes within the NEC intestine suffer impaired migratory capacity due to abnormal induction of autophagy [91]. The MSP-RON signaling pathway is a critical developmental pathway within the epithelium, and complete disruption of RON is embryonically lethal [92]. MSP-RON directs EMT [93] and has been shown to play a role in modulating inflammation and epithelial repair in the gut [94]. Activated by MSP, RON, a receptor tyrosine kinase primarily expressed in epithelial cells, attenuates inflammation by inhibiting nitric oxide and pro-inflammatory cytokine production through PI3K/Akt and RAS/ERK/MAPK pathways [95]. High RON expression within the epithelium suggests a crucial role in maintaining tissue integrity and repair rather than direct modulation of immune responses. Interestingly, single nucleotide polymorphism variants such as 689C within the MSP gene, reducing MSP levels, are associated with an increased risk of intestinal inflammatory diseases and impaired wound healing [96].

Our results indicate HA35 treatment enriches the MSP-RON pathway in the terminal ileum, and parallels similar findings in the lung. In the latter tissue, LMW HA interacts with receptor for hyaluronan-mediated mobility (RHAMM) and RON to enhance mucociliary function and clearance of pathogens [97]. Whether such interactions among HA35, RHAMM, and RON occur in the developing neonatal intestinal epithelium or contribute to repair of wounds incurred during NEC is not yet clear.

### 3.6. The Role of HA35 as a Developmentally Appropriate Oncofetal Signal

Oncogenes play a significant role in embryonic, fetal, and adult tissues, promoting rampant proliferation of cells during development or repair [98]. While most commonly associated with carcinogenic and metastatic processes, oncogene activity during healthy development is tightly regulated to promote maximal proliferation within the bounds of healthy tissue growth. Fetal stem cell programs can be induced within the intestine via a variety of extrinsic factors [99], and we propose oral supplementation with HA35 through provision of HM is one such extrinsic factor. Within our dataset, multiple oncogenes and proto-oncogenes (e.g., *Kras*, *Mdm2* [a key regulator of the tumor suppressor, p53], *Rala* [RAS-like proto-oncogene], *Lmo4* [LIM domain only 4]), and oncogenic pathways (e.g., HER-2, 14-3-3, MSP-RON, ErbB signaling) are upregulated in the ileum of mouse pups treated with HA35, and the collective effect of these synchronized cues induces maturation and development of the neonatal murine intestine [38].

The content of GAGs and HA, in particular, within term and preterm milk declines over the first six months of breastfeeding [100] from a peak in colostrum shortly after birth [29]. Interestingly, as with other GAGs [101], HA35 content is significantly elevated in the HM provided to preterm compared with term infants (unpublished data). We posit that HA35 serves as an oncofetal signal within the infant intestine, and its maximal content in preterm colostrum is consistent with an essential role in development and maturation of the underdeveloped preterm intestine. As with other instances of oncogene expression within healthy tissue, provision of the oncofetal HA35 is tightly regulated via maternal mammary gland production and subsequent feeding events. Importantly, the duration of maximal HA35 content within preterm HM overlaps with the period of enhanced susceptibility to NEC [46].

## 4. Materials and Methods

### 4.1. Hyaluronic Acid 35 kDa (HA35)

HA35 was purchased from Lifecore Biomedical (Chaska, MN, USA). HA35 is a biosynthetic and medical grade polydisperse mixture of HA from 21 kDa to 40 kDa, with an average MW of 35 kDa. The compound is pure and commercially manufactured to meet the European Pharmacopoeia and Japanese Pharmacopoeia monographs for sodium hyaluronate.

### 4.2. Mouse Experiments

All animal studies were approved by the Institutional Animal Care and Use Committee at the University of Oklahoma Health Sciences Center (IACUC #19-062-EFCHI). Data reported here result from additional analysis of an animal experiment originally conducted and published in 2021 [38]. Briefly, timed pregnant CD-1 dams (Charles River Laboratories, Wilmington, MA, USA) were housed individually in sterilized, filter-top cages. Ad libitum food and water were provided under a 12 h light/dark cycle. Pups of both sexes were born vaginally, weighed daily, and randomized into groups receiving 30 mg/kg HA35 or control (an equal volume of sterile phosphate-buffered saline) via oral gavage from postnatal day (P)7 to P14. At treatment end, pups from both groups were anesthetized with isoflurane and euthanized via bilateral thoracotomy and cardiac puncture. Terminal ileum was harvested and stored at −80 °C in RNAlater (Invitrogen, Carlsbad, CA, USA) for downstream gene analyses.

### 4.3. Bulk RNA-Sequencing

Ileal tissues were homogenized with a 7 cm polypropylene pellet pestle (W.W. Grainger, Lake Forest, IL, USA) and passed through a QIAshredder homogenizer (Qiagen, Germantown, MD, USA). Total RNA was isolated using a RNeasy Plus Mini Kit (Qiagen). Sample quantity and quality were evaluated using a NanoDrop Lite Spectrophotometer (ThermoFisher Scientific, Waltham, MA, USA). Stranded RNA-Seq libraries were constructed for 8 animals via the CORALL Total RNA-Seq Library Prep Kit v2 (Lexogen, Greenland, NH, USA). Libraries were constructed with 200–500 ng of RNA. Libraries were indexed for multiplex sequencing, pooled, and run on the NovaSeq 6000 platform (Illumina, San Diego, CA, USA) at the Oklahoma Medical Research Foundation Clinical Genomics Center. Sequences were analyzed through a custom computational pipeline consisting of the open-source gSNAP, Cufflinks, and R for sequence alignment and evaluation of differential gene expression [102]. Reads generated were mapped to the mouse genome (mm10) by gSNAP [103], expression (fragments Per Kilobase of transcript per Million mapped reads, FPKM) derived by Cufflinks [104], and differential expression analyzed using R.

### 4.4. Analysis of Differentially Expressed Genes (DEGs)

DEGs with a *p*-value < 0.05 and a |Log2FoldChange (FC)| > 1 were collated using DeSeq2 in R. Significant genes were visualized with one-way hierarchical heatmaps using the ‘heatmap.2’ function in the R gplots package.

### 4.5. Bioinformatic Analysis of DEGs

DEGs from all 8 samples were subjected to principal component analysis (PCA) using the prcomp function and visualized by autoplot using the ggfortify package in R.

Cell deconvolution for mixed cell types within the small intestine, as well as intestinal epithelial-specific cell types, was performed using the granulator package in R and expression matrices derived from the scRNA-seq Mouse Cell Atlas [40]. Median cell abundances were compared using Wilcoxon signed rank test and cell proportions were plotted using stacked box plots.

### 4.6. Protein–Protein Interaction (PPI) Networks

*p*-values and FC values for DEGs were imported to CytoScape v3.10.2 [105]. Overlapping gene–protein interaction networks were constructed via query of the STRING (search tool for the retrieval of interacting genes) database (confidence cutoff, 0.4) using the Cytoscape stringApp [106], where nodes indicate discrete genes and edges represent connections between genes. The largest subnetwork was selected for each analysis, and annotation of functional enrichment was performed with ClueGO [107], using GO (gene ontology) Biological Functions, GO Immune System, GO Molecular Function, KEGG (Kyoto Encyclopedia of Genes and Genomes), Reactome Pathways, and WikiPathway databases. Significantly enriched functions were defined through two-sided hypergeometric tests with Bonferroni step down correction (Bonferroni-corrected *p* < 0.05), with a minimum of 2–3 genes/pathway and a kappa score of 0.4. Hub genes were identified through degree of connectivity and shortest paths using maximal clique centrality (MCC) ranking of 15 or fewer nodes in the CytoScape plugin, CytoHubba [108].

### 4.7. Ingenuity Pathway Analysis (IPA)

DEGs with *p* < 0.05 were uploaded into IPA (Qiagen) for pathway analysis. Significantly altered or enriched pathways and predicted upstream regulators were defined by significant Z-scores determining the probability of association between genes in the dataset and a pathway of interest compared with chance alone. Core Analysis was used to explore differential biological processes, canonical pathways, upstream transcriptional regulators, and gene networks. Significantly enriched or depleted pathways (FDR-adjusted *p* < 0.05 via Fisher’s Exact Test) with gene overlap ≥2 were represented with bubble plots using ggplot2 and dplyr packages in R. IPA Functional Analysis was used to identify biological functions and/or diseases significantly associated with the dataset. To assess the activation state of a pathway and upstream regulators, the consistency of the match between the observed and predicted pattern was calculated (Z-score). A positive Z-score signifies a regulator in the active state, while a negative Z-score indicates repressed activity. Canonical pathway analysis was used to identify networks from the IPA library most significantly modulated across both groups. The significance of the association between each dataset and the canonical pathway was measured as a ratio of the number of molecules from the dataset mapped to the pathway divided by the total number of molecules mapped to the canonical pathway.

### 4.8. GSEA Analysis

Gene set enrichment analysis (GSEA) was conducted with GSEA 4.1.0 against the Hallmark database, and significant pathways were defined as FDR-adjusted *p* < 0.05 and gene overlap > 15.

## 5. Conclusions

HA35 significantly influences gene expression related to small intestinal development and maturation. The observed upregulation of pathways associated with cellular growth, differentiation, immune regulation, and response to physiological stressors, coupled with downregulation of apoptotic signaling, underscores the potential of HA35 as a HM bioactive factor capable of lending protection against NEC. Notably, HA35 upregulates genes incorporated in the KEAP1/NFE2L2 and mTOR signaling pathways, known to support antioxidative responses and cellular growth, respectively. These pathways are relevant in the context of NEC, as oxidative stress and impaired cellular repair mechanisms are critical in the progression of, and possible healing from, this disease. Confirmation of our findings will now proceed in human preterm infant intestinal epithelial enteroid models.

## Data Availability

The original sequencing data presented in this study are available in the GEO database (accession number GSE274261), with public access available from 31 December 2024.

## Supplementary Materials

The following supporting information can be downloaded at: https://www.mdpi.com/article/10.3390/ijms252312679/s1.
