# Supplementary material for: Network-Based Bioinformatics Highlights Broad Importance of Human Milk Hyaluronan
Source: Int J Mol Sci. 2024 Nov 26;25(23):12679. doi: 10.3390/ijms252312679 (PMC11641400; doi:10.3390/ijms252312679)
Supplement: Supplementary file 1 [file ijms-25-12679-s001.zip › Figure S1.pdf]

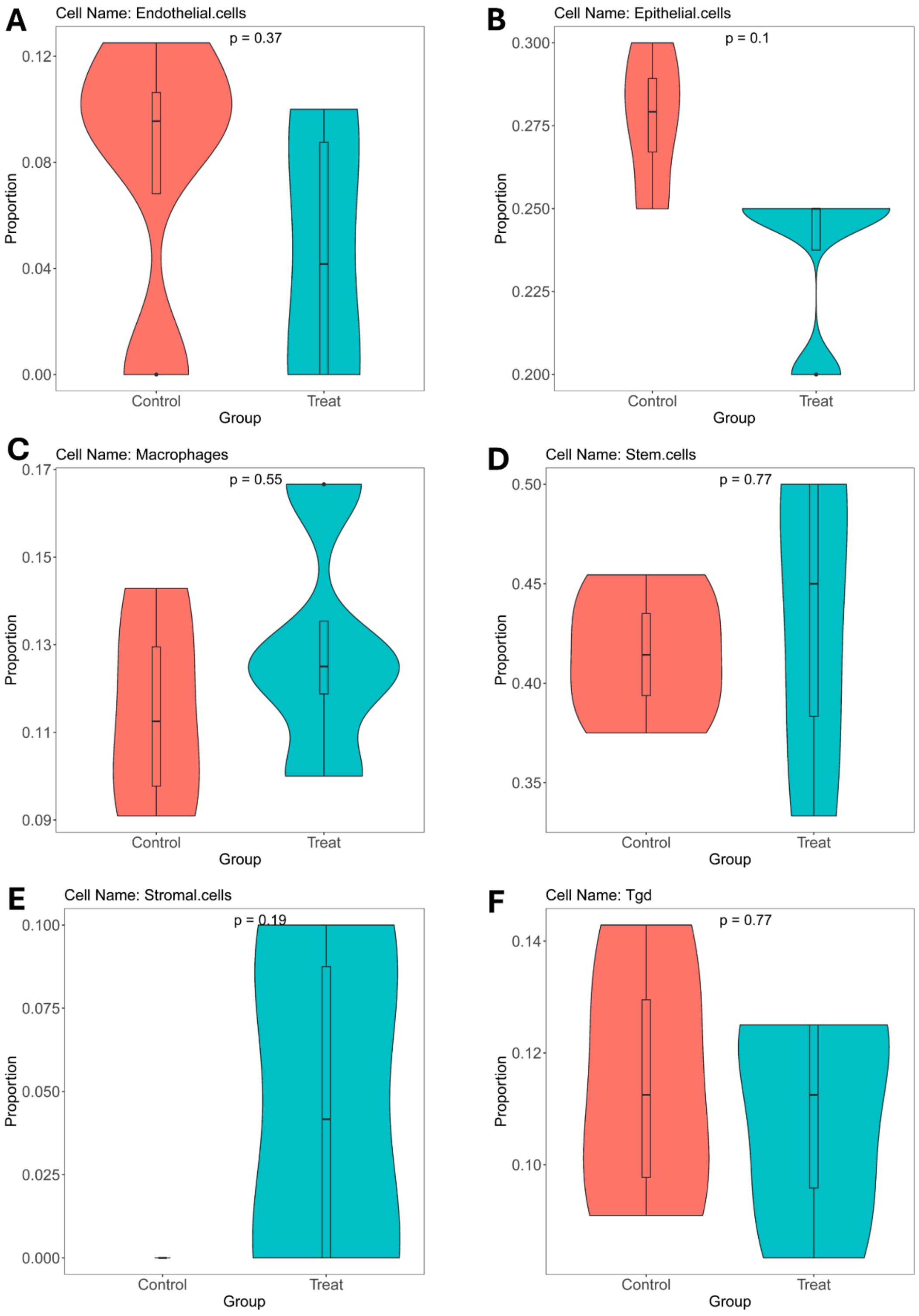

Supplementary Figure S1. Cell deconvolution results for **(A)** endothelial cells, **(B)** epithelial cells, **(C)** macrophages, **(D)** stem cells, **(E)** stromal cells, and **(F)** gamma delta T cells in control and HA35-treated pup ileum.
