# Supplementary material for: Network-Based Bioinformatics Highlights Broad Importance of Human Milk Hyaluronan
Source: Int J Mol Sci. 2024 Nov 26;25(23):12679. doi: 10.3390/ijms252312679 (PMC11641400; doi:10.3390/ijms252312679)
Supplement: Supplementary file 1 [file ijms-25-12679-s001.zip › Figure S2.pdf]

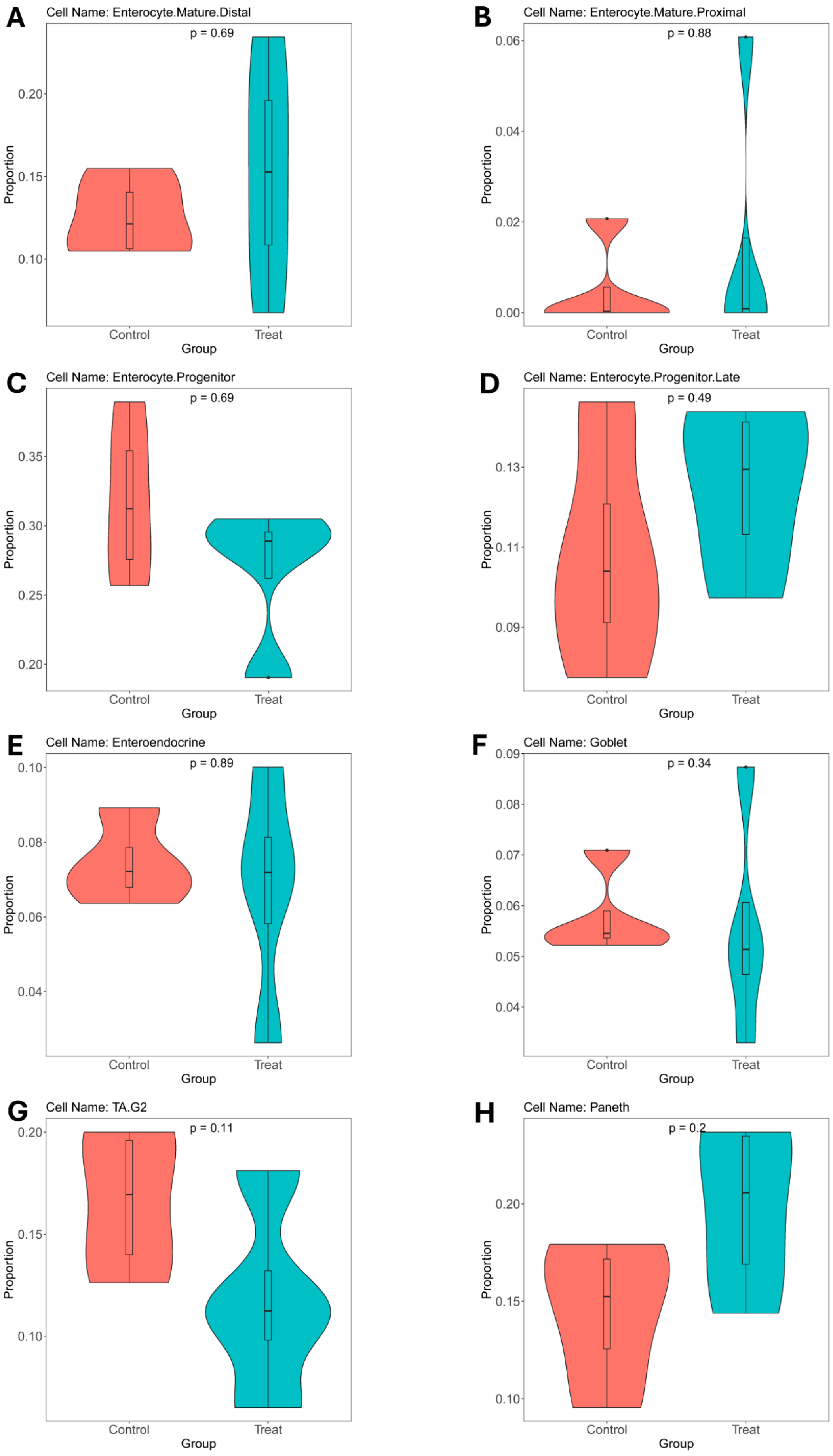

Supplementary Figure S2.

Cell deconvolution results for **(A)** mature distal enterocytes, **(B)** mature proximal enterocytes, **(C)** enterocyte progenitor, **(D)** late enterocyte progenitor, **(E)** enteroendocrine, **(F)** goblet, **(G)** transit-amplifying G2, and **(H)** Paneth cells in control and HA35-treated pup ileum.
