# Supplementary material for: Network-Based Bioinformatics Highlights Broad Importance of Human Milk Hyaluronan
Source: Int J Mol Sci. 2024 Nov 26;25(23):12679. doi: 10.3390/ijms252312679 (PMC11641400; doi:10.3390/ijms252312679)
Supplement: Supplementary file 1 [file ijms-25-12679-s001.zip › Figure S3.pdf]

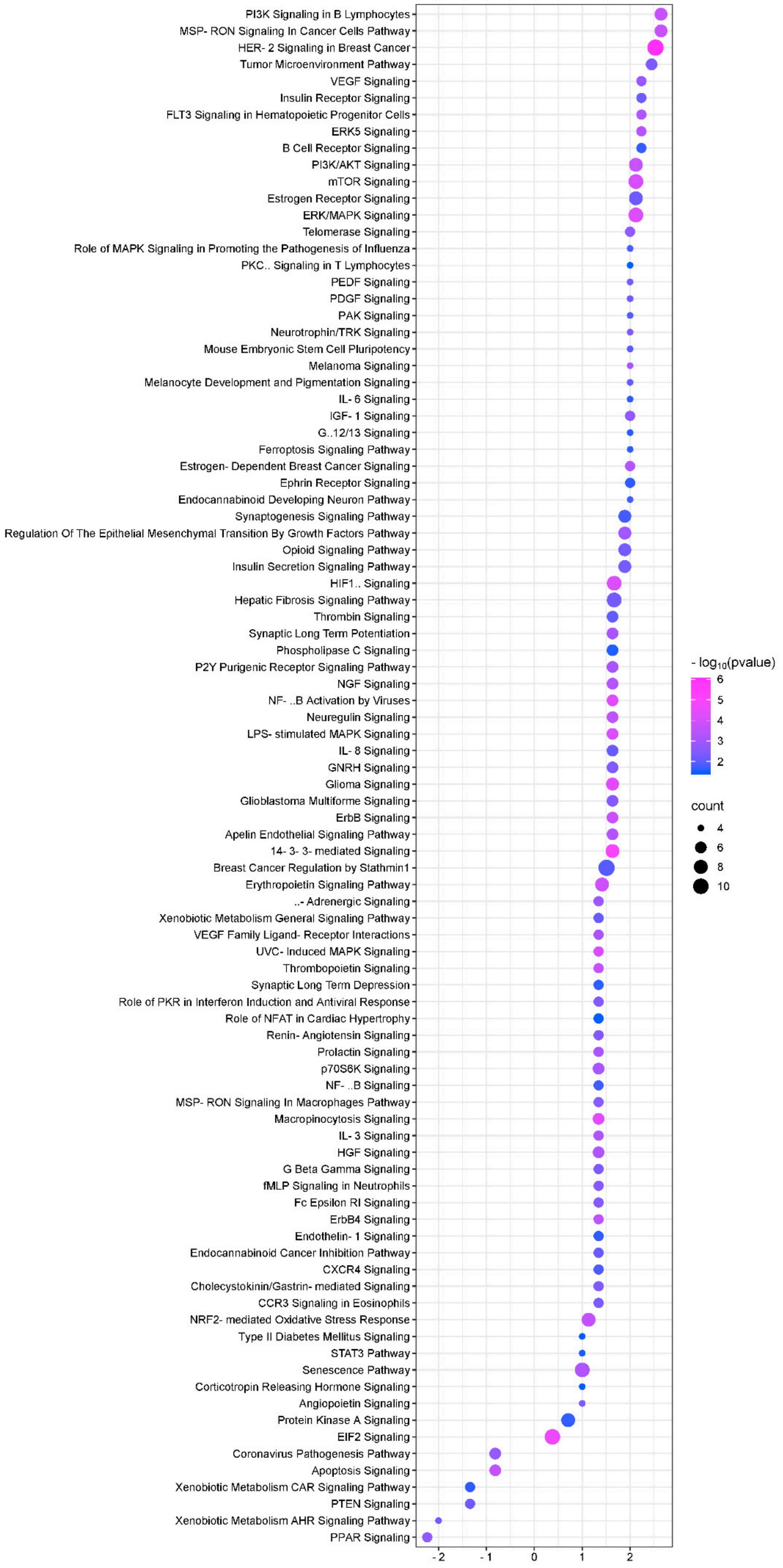

Supplementary Figure S3. Full

bubble plots for Ingenuity Pathway Analysis enriched and depleted pathways in HA35-treated vs. control P14 mouse pup ileum.
